# Secular changes in the progression of clinical markers and patient-reported outcomes in early rheumatoid arthritis

**DOI:** 10.1093/rheumatology/kez635

**Published:** 2020-01-03

**Authors:** Lewis Carpenter, Elena Nikiphorou, Patrick D W Kiely, David A Walsh, Adam Young, Sam Norton

**Affiliations:** 1 Health Psychology Section, King’s College LondonUK; 2 Centre for Rheumatic Diseases, King’s College LondonUK; 3 Department of Rheumatology, St George’s University Hospital NHS Foundation TrustLondon, UK; 4 Institute of Medical and Biomedical Education, St George's University of London, LondonUK; 5 Arthritis UK Pain Centre, University of Nottingham, Nottingham, UK; 6 Postgraduate Medicine, University of Hertfordshire, Hatfield, UK

**Keywords:** inflammatory arthritis, cohort studies, longitudinal analysis, patient-reported outcomes

## Abstract

**Objectives:**

To examine secular trends in the progression of clinical and patient-reported outcomes in early RA.

**Methods:**

A total of 2701 patients recruited to the Early Rheumatoid Arthritis Study or Early Rheumatoid Arthritis Network with year of diagnosis from 1986 to 2011. The 5-year progression rates for patients diagnosed at different points in time were modelled using mixed-effects regression; 1990, 2002 and 2010, were compared. Clinical markers of disease included the 28-joint count DAS and the ESR. Patient-reported markers included the HAQ, visual analogue scale of pain and global health, and the Short-Form 36.

**Results:**

Statistically significant improvements in both 28-joint count DAS and ESR were seen over the 5 years in patients diagnosed with RA compared with those diagnosed earlier. By 5 years, 59% of patients with diagnosis in 2010 were estimated to reach low disease activity compared with 48% with diagnosis in 2002 and 32% with diagnosis in 1990. Whilst HAQ demonstrated statistically significant improvements, these improvements were small, with similar proportions of patients achieving HAQ scores of ≤1.0 by 5 years with a diagnosis in 1990 compared with 2010. Levels of the visual analogue scale and the Mental Component Scores of the Short-Form 36 indicated similar, statistically non-significant levels over the 5 years, irrespective of year diagnosed.

**Conclusion:**

This study demonstrates improvements in inflammatory markers over time in early RA, in line with improved treatment strategies. These have not translated into similar improvements in patient-reported outcomes relating to either physical or mental health.

**Rheumatology key messages**

- Changes in the presentation and clinical management of RA has seen improvements in inflammation.
- These improvements in inflammation, however, are not translating into improvements in patient-reported outcomes.
- More emphasis needed on mental health, pain and fatigue in research and clinical care.

## Introduction

The last 30 years have seen many changes in the presentation of RA in the clinics, as well as how it is managed therapeutically. Recent data from early RA cohorts highlight how new RA patients are presenting with increased levels of comorbidities and higher levels of obesity [1], as well as increased levels of patient-reported outcomes (PROs), such as pain and fatigue [2]. Alongside these changes in clinical presentation, a number of significant changes in the therapeutic management of RA has also taken place. This includes the switch to MTX as the anchor DMARD, the introduction of biologic DMARDs (bDMARDs) and the adoption of a treat-to-target approach [3, 4].

There is growing evidence that these therapeutic changes have had positive effects on lowering inflammation and halting the progression of structural joint damage [5–7]. Data from the Norfolk Arthritis Register (NOAR) demonstrated that patients recruited between 2000 and 2004 had significant improvements in disease activity compared with those patients recruited between 1990 and 1994 [8]. However, there was little difference in functional disability, and therefore it is unclear whether these improvements in disease activity have translated into improvements in key PROs, such as mental health, fatigue and pain [9–12].

Studies have shown that psychological distress, including depression and anxiety, is more prevalent in patients living with RA [13–16], although its precise relationship with disease activity is not clear [17]. A recent systematic review highlighted how health-related quality of life (HRQoL) was reduced in RA populations, with measures of physical function, bodily pain, fatigue and mental well-being lower than that of the UK and US general population [15]. Furthermore, health economic evaluations have shown that those RA patients with worse HRQoL outcomes are associated with higher health care resource utilization [18]. Whilst few in number, longitudinal data from observational studies have also sought to examine the long-term progression of HRQoL outcomes for early RA patients. One study showed that levels of psychological well-being and functional disability remained relatively stable over a 10-year period [19], whilst a study of a small cohort of early RA patients in Sweden indicated greater improvements in physical and mental health for both men and women over a 6-year period [20].

Previous studies examining secular changes in HRQoL outcomes have primarily focussed on functional disability using the HAQ, as this is a common outcome measure in arthritis. The current evidence base suggests that whilst patients diagnosed and treated in earlier cohorts demonstrate statistically significant differences in HAQ, these changes relate to small absolute differences in the overall score [8, 11]. Despite reductions in these HRQoL outcomes, these patients still exhibited pronounced levels of pain and disability compared with reference values [11].

To date, there has been no study utilizing longitudinal analytical techniques to assess secular trends in long-term trajectories of pain, functional disability and HRQoL in early RA patients diagnosed over a 30-year time frame. This study examines progression of disease activity, functional disability and measures of HRQoL from two early longitudinal RA cohorts, recruiting between 1986 and 2011. Longitudinal data for both cohorts allows for the estimation of 60-month trajectories over different time periods. It is hypothesized that 60-month trajectories of disease activity and other objective markers of inflammation have seen improvements in more recent decades, but functional disability, pain and HRQoL remain largely unchanged.

## Methods

### Patients

The data used for this study were collected from two longitudinal inception cohorts: the Early Rheumatoid Arthritis Study (ERAS) and the Early Rheumatoid Arthritis Network (ERAN). ERAS recruited 1465 patients from across the UK between 1986 and 2001, while ERAN recruited 1236 patients from across the UK between 2002 and 2011. All patients had a confirmed diagnosis of RA and were recruited within 2 or 3 years of symptom onset, typically prior to conventional DMARD initiation. Maximum follow-up for ERAS was 25 years (median 10 years) and for ERAN was 11 years (median 3 years). Standard clinical, laboratory and radiographic data were collected at baseline, 6 months and 12 months, and then yearly thereafter.

### Treatment

All patients were treated based on standard clinical practice at the time [21]. For ERAS, this typically meant DAMRD monotherapy, largely SSZ, with a gradual switch to MTX over time [22]. For ERAN, SSZ and MTX were used in equal proportions, with a shift to predominately MTX towards the end. Median time to first DMARD was 2 months for ERAS and 1 month for ERAN. All patients in ERAS were DMARD naïve, whereas in ERAN a small proportion (13.5%) had commenced DMARD therapy prior to baseline visit. Combination DMARD therapies were reserved for more severe disease (around 25%) and, from 2002, <10% of patients received bDMARDs by 3 years.

### Measures

#### Disease activity score

For ERAS, the original three variable 44-joint DAS (DAS44) was used to measure disease activity, comprised of the 44 swollen joint count, Ritchie Index for tender joint count and ESR. For ERAN, the 4-variable 28-joint count DAS (DAS28) was used, comprised of the revised 28 swollen joint count and tender joint count, ESR and a Patient Global Assessment (PGA). For those where ESR was missing, but a value of CRP was available, the DAS28-CRP version was used [23]. To enable comparison of disease activity across the two cohorts, the DAS44 in ERAS was converted to DAS28 using a recently developed transformation formula that has been validated in the ERAS cohort [24].

#### Functional disability

The UK version of the HAQ disability index was used to collect data on patient’s functional disability [25]. Consisting of 20 items across eight domains of daily living, it provides an overall disability score that ranges from 0 to 3. Generally, a score of >1 indicates moderate disability, whilst scores >2 indicate more severe disability.

#### Visual analogue scale

For patients recruited into ERAN, the PGA was recorded, which is a sub-component of the DAS28 and asks patients to rate their overall health on a visual analogue scale (VAS) from 0 to 100. However, patients recruited to ERAS did not record PGA (except in one centre), but instead rated current pain levels using a 0–100 VAS.

Whilst the focus of the item is specifically on pain for ERAS, using a small subset of patients (*n* = 85) with both the PGA and pain VAS, it was found that both scores correlated highly (*r*_ICC_ = 0.9, *P* < 0.001), with Bland and Altman plots indicating only a 0.39 mean difference (95% limits of agreement –23.57 to 22.80) between the scores. As such we decided it was appropriate to compare changes in VAS over time across both cohorts [26].

#### Short-Form 36

The Short-Form 36 (SF-36) was used to assess patients’ quality of life for ERAN patients only. Patients are assessed on quality of life across eight domains; Physical Function, Physical Role, Vitality, Mental Health, Emotional Role, Bodily Pain, General Health and Social Functioning. Two summary component scores are calculated, the Physical Component Summary (PCS) and Mental Component Summary (MCS). Scores are normalized to the UK national average [27], whereby 50 indicates the population average score and a difference of 10 units indicate 1 s.d. difference in the general population (e.g. ∼16% of the general population score <40, and 2% <30). The overall MCS and Mental Health sub-domain will be used as measures of mental well-being, whilst the Vitality sub-domain will be used as a measure of fatigue.

#### Other clinical measures

Seropositivity was assessed using RF and for a subset of patients recruited after 2000 anti-CCP was also recorded. Patients who were positive on either RF or anti-CCP were defined as seropositive, whilst those that indicated negative to both were defined as seronegative. ESR was recorded at each follow-up and used as an objective marker of inflammation, along with CRP, which was available in 46% of patients. Data on comorbidities were recorded at each clinical visit and were coded according to the 10th revision of the International Classification of Diseases. These codes were used to generate a weighted Charlson Comorbidity Index [28]. The score was modified to remove RA, as it was the index condition for this study.

### Statistical analysis

Descriptive analysis for all variables were explored for both ERAS and ERAN to determine differences in demographic, clinical or laboratory data. Means and s.d., or medians and interquartile range were used where appropriate depending on the distribution of the underlying data. For categorical data, number of patients and proportions of total cohort (excluding missing data) were provided.

To examine the rate of progression of the DAS28, HAQ, VAS, ESR and SF-36 over 5 years of follow-up, mixed-effects linear regression models were used. The analyses were restricted to 5 years since the rate of attrition was high beyond this point in the ERAN cohort. Mixed-effects regression models with a random intercept allows for the non-independence of data to be accounted for, whereby each patient had repeated observations over time. Preliminary analyses identified each of the outcomes to have a non-linear progression over the 5 years, indicating a greater change in the first 12 months (the initial treatment response), with a more gradual change from months 12–60. To account for this, months from baseline assessment was included in the model as linear splines with knots at both 6 and 12 months. The models also controlled for important confounding factors, including age at disease onset, gender, seropositivity, baseline BMI, baseline Charlson Comorbidity Index, and DMARD or steroid use prior to baseline visit. The calendar year in which the patient was diagnosed was entered as a main effect and with an interaction term with month of follow-up, in order to allow for different rates of change over time between the different calendar periods. The model allowed for estimated mean scores to be calculated at different years of diagnosis, over the 60-month follow-up period. These were used to display the trends over time graphically for a number of selected dates; 1990, 2002 and 2010, along with corresponding 95% CIs. These dates represented the early, middle and late end of both cohorts combined.

Further analysis dichotomized each outcome to determine the proportion of patients achieving pre-specified ‘good’ outcomes by 60 months. DAS28 was based on whether they achieved low disease activity (LDA) (DAS28 ≤3.2), ESR and PGA were based on achieving ≤10 units, reflecting Boolean remission critiera [29], HAQ was based on achieving a score ≤1 [30], and SF-36 PCS and MCS were based on achieving scores ≥50 units, which reflects the UK population average. The probability of achieving low scores over the 60 months was estimated using a mixed-effects logistic regression analysis, following a similar modelling method as the linear models described above. All analyses were conducted using Stata (StataCorp. 2017. *Stata Statistical Software: Release 15*. College Station, TX: StataCorp LLC.) and significance was assumed at *P* < 0.05.

## Ethics

The ERAS study received ethical approval from the West Hertfordshire Local Research Ethics Committee and subsequently from the Caldicott Guardian. The ERAN study was approved by Trent Research Ethics Committee (reference 01/4/047). All participants gave signed, informed consent to participate in line with the Declaration of Helsinki.

## Results

A summary of the demographic, and baseline clinical and laboratory variables is shown in Table 1. Patients in ERAN were older at onset and more likely to be female. Mean baseline DAS28, VAS and HAQ levels were similar across the two cohorts, whilst patients in ERAN had lower mean levels of ESR and HAQ, along with a smaller proportion of patients with seropositive RA.

**Table 1:** Baseline demographic and clinical variables

	Total *N =* 2701	ERAS *N* = 1465	ERAN *N* = 1236
Years recruited			
Range	1986–2011	1986–2001	2002–2011
Age at onset			
*N* (missing)	2701 (0)	1465 (0)	1236 (0)
Mean (s.d.)	56.1 (14.43)	55.3 (14.57)	57 (14.22)
Median (IQR)	57 (46.0–67.0)	57 (45.0–66.0)	58 (47.0–68.0)
Range	0–93	17–93	0–89
Female			
*N* (%)	1812 (67.09)	973 (66.42)	839 (67.88)
Missing (%)	0 (0)	0 (0)	0 (0)
Baseline BMI			
*N* (Missing)	2392 (309)	1272 (193)	1120 (116)
Mean (s.d.)	26.5 (5.00)	25.6 (4.50)	27.6 (5.30)
Median (IQR)	25.9 (23.0–29.2)	25 (22.5–28.0)	26.8 (23.9–30.5)
Range	14–55	15–49	14–55
Baseline Charlson Comorbidity Index			
*N* (missing)	2698 (3)	1465 (0)	1233 (3)
Mean (s.d.)	0.2 (0.61)	0.1 (0.38)	0.4 (0.80)
Median (IQR)	0 (0.0–0.0)	0 (0.0–0.0)	0 (0.0–0.0)
Range	0–7	0–3	0–7
Baseline DAS28			
*N* (missing)	2588 (113)	1399 (66)	1189 (47)
Mean (s.d.)	4.8 (1.47)	5 (1.35)	4.6 (1.58)
Median (IQR)	4.9 (3.8–5.9)	5 (4.1–6.0)	4.7 (3.5–5.7)
Range	0–9	1–8	0–9
Baseline pain VAS			
*N* (missing)	2642 (59)	1411 (54)	1231 (5)
Mean (s.d.)	43.8 (26.01)	44 (26.37)	43.5 (25.61)
Median (IQR)	45 (23.0–63.0)	45 (23.0–63.0)	45 (22.0–63.0)
Range	0–100	0–98	0–100
Baseline ESR			
*N* (missing)	2511 (190)	1458 (7)	1053 (183)
Mean (s.d.)	37.2 (27.52)	42.2 (28.79)	30.2 (24.00)
Median (IQR)	30 (15.0–54.0)	37 (18.0–62.0)	24 (12.0–41.0)
Range	1–140	1–140	1–126
Baseline HAQ			
*N* (missing)	2660 (41)	1460 (5)	1200 (36)
Mean (s.d.)	1.1 (0.77)	1.1 (0.77)	1.1 (0.76)
Median (IQR)	1 (0.5–1.6)	1 (0.5–1.8)	1 (0.5–1.6)
Range	0–3	0–3	0–3
Seropositive			
*N* (%)	1569 (62.04)	914 (62.77)	655 (61.04)
Missing (%)	172 (6.37)	9 (0.61)	163 (13.19)

Baseline demographic and clinical variables for all patients and stratified by the separate ERAS and ERAN cohorts. DAS28: 28 joint count DAS; ERAN: Early Rheumatoid Arthritis Network; ERAS: Early Rheumatoid Arthritis Study; IQR: interquartile range; VAS: visual analogue scale.

In order to account for these differences in baseline characteristics, all analyses controlled for age at disease onset, gender, seropositivity status, baseline BMI and baseline Charlson Comorbidity Index, along with steroid or DMARD use prior to baseline visit.

### Measures of disease activity and inflammation

**Fig. 1:**
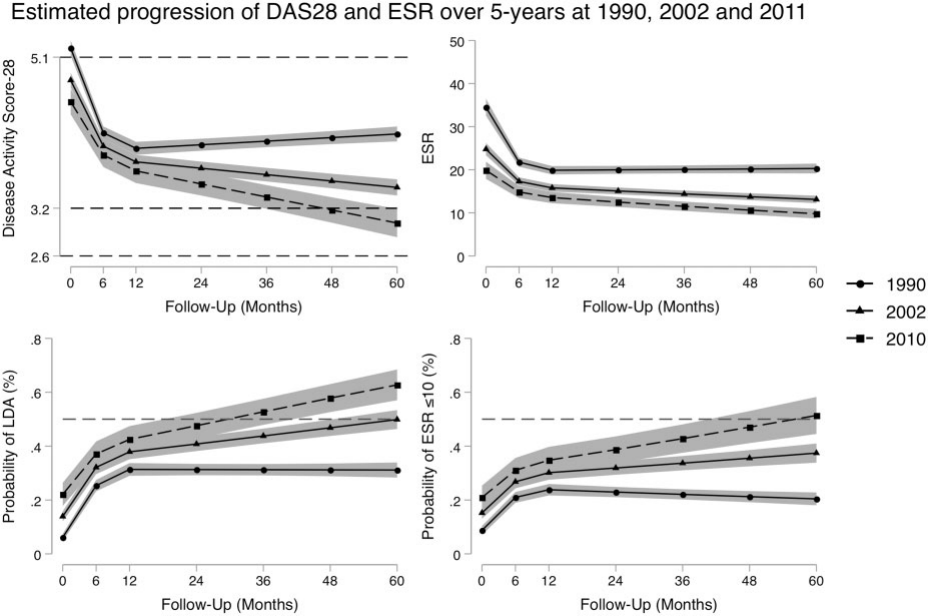
Estimated mean scores of the DAS28 and ESR scores, along with the predicted probability of achieving LDA and a ESR ≤10 over the first 60 months for patients diagnosed in 1990, 2002 and 2010 For the DAS28, black dotted lines indicate thresholds at 2.6 for remission, 3.2 for LDA and 5.1 for HDA. Shaded areas represent the 95% CIs. Patients were diagnosed in: 1990 (circle markers solid line), 2002 (triangle marker solid line) or 2010 (square marker dashed line). For the probability graphs, the red dotted line indicates the 50% probability level. Models controlled for age at onset, gender, seropositivity status at baseline, baseline BMI, baseline Charlson Comorbidity Index, and use of DMARDs and steroids at baseline assessment. DAS28: 28-joint count DAS; LDA: low disease activity; HDA: high disease activity.

Mixed-effects linear regression models were used to assess the progression of the DAS28 and log-transformed ESR over the first 5 years of the patient’s disease, with estimated mean scores for patients diagnosed in 1990, 2002 and 2010. These are shown in Fig. 1. Comparisons in the estimated mean scores at baseline and at month 60 between patients diagnosed in 2010 and 1990, and between patients diagnosed in 2010 and 2002, are illustrated in Table 2. At baseline, there were statistically significant differences between the three time periods, and these were increased by month 60, where those diagnosed in 2010 had statistically significantly improved DAS28 scores of 1.12 units (95% CI 0.90, 1.35) compared with 1990 (*P* < 0.001) and of 0.45 units (95% CIs 0.36–0.54) compared with 2002 (*P* < 0.001).

**Table 2:** Estimated differences of each outcome at baseline and 6-months between 2010, 2002 and 1990

	DAS28				ESR			
	2010 *vs* 1990		2010 *vs* 2002		2010 *vs* 1990		2010 *vs* 2002	
	Delta	95% CI	Delta	95% CI	Delta	95% CI	Delta	95% CI
Baseline	−0.68***	−0.88, 0.47	−0.27***	−0.35, 0.19	−14.62***	−17.80, −11.43	−4.90***	−5.76, −4.04
Month 60	−1.12***	−1.35, −0.90	−0.45***	−0.54, −0.36	−10.49***	−12.30, −8.67	−3.31***	−3.673, −2.89
	HAQ				VAS			
	2010 *vs* 1990		2010 *vs* 2002		2010 *vs* 1990		2010 *vs* 2002	
	Delta	95% CI	Delta	95% CI	Delta	95% CI	Delta	95% CI
Baseline	−0.15*	−0.26, −0.05	−0.06*	−0.10, −0.02	−1.20	−4.570, 2.30	−0.48	−1.88, 0.92
Month 60	−0.24**	−0.36, −0.13	−0.10**	−0.14, −0.05	−0.35	−4.12, 3.42	−0.14	−1.65, 1.37

The estimated mean differences, along with their corresponding 95% CI, for the DAS28, ESR, HAQ and VAS at baseline and at month 60 comparing patients diagnosed in 2010 with those diagnosed in 1990, and those diagnosed in 2010 with those diagnosed in 2002. Models controlled for age at onset, gender, seropositivity status at baseline, baseline BMI, baseline Charlson Comorbidity Index, and use of DMARDs and steroids at baseline assessment.

* *P* < 0.05,

** *P* < 0.01,

*** *P* < 0.001.

DAS28: 28 joint count DAS; VAS: visual analogue scale.

This was also reflected in the mixed-effects logistic regression model investigating the probability of achieving LDA over the 60 months. Whilst it is estimated that ∼31% of patients reached LDA by 60 months where they were diagnosed in 1990 [odds ratio (OR) 0.31; 95% CI 0.28, 0.34], 50% reached LDA in where they were diagnosed in 2002 (OR 0.50; 95% CI 0.46, 0.53), and 63% were estimated to reach LDA if they were diagnosed in 2010 (OR 0.63; 95% CI 0.57, 0.68).

The declines in DAS28 were in part due to reductions in ESR levels, where patients diagnosed in 2010 had significantly lower ESR at baseline and at month 60 relative to those diagnosed in 1990 (*P* < 0.001) and 2002 (*P* < 0.001) (Fig. 1 and Table 2). This was also evident in the logistic regression model, which estimated the probability of achieving a ESR ≤10, where 51% (OR 0.51; 95% CI 0.45, 0.58) of patients diagnosed in 2010 were estimated to reach ESR levels ≤10, compared with 37% (OR 0.37; 95% CI 0.34, 0.41) in 2002 and 20% (OR 0.20; 95% CI 0.18, 0.23) in 1990.

### Measures of patient-reported outcomes

**Fig. 2:**
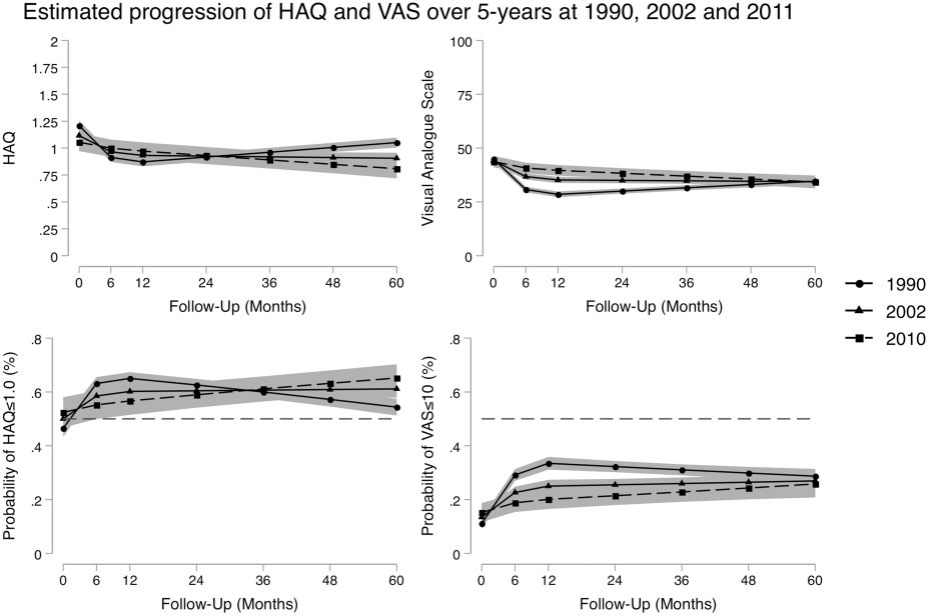
Estimated marginal means of the functional disability (HAQ) and VAS scores, along with the predicted probability of achieving a HAQ ≤1 or a VAS of ≤10 units over the first 60 months for patients diagnosed in 1990, 2002 and 2010 Patients were diagnosed in: 1990 (circle markers solid line), 2002 (triangle marker solid line) or 2010 (square marker dashed line). Shaded areas represent the 95% CIs. For the probability graphs, the red dotted line indicates the 50% probability level. Models controlled for age at onset, gender, seropositivity status at baseline, baseline BMI, baseline Charlson Comorbidity Index, and use of DMARDs and steroids at baseline assessment. VAS: visual analogue scale.

The results of the estimated mean scores for HAQ and VAS from the mixed-effects linear models are given in Fig. 2 and presented in Table 2. Whilst the models indicated statistically significant improvements at baseline and at month 60 between patients diagnosed in 2010 and 1990 (*P* < 0.05) and between those diagnosed in 2010 and 2002 (*P* < 0.05) for both outcomes, these differences were small. This is reflected in the logistic regression models looking at the probability of achieving HAQ scores ≤1.0 and VAS scores ≤10 units, where by month 60 the probability for HAQ was similar across the recruitment periods. Some 54% of patients indicated a HAQ ≤1 at 60 months where they were diagnosed in 1990 (OR 0.54; 95% CI 0.51, 0.57), compared with 61% of patients who were diagnosed in 2002 (OR 0.61; 95% CI 0.58, 0.64) and 60% of patients diagnosed in 2010 (OR 0.65; 95% CI 0.60, 0.70) (Fig. 2). The VAS differed from other PROs in that it indicated a statistically significant improvement for those patients diagnosed in 1990 relative to 2002 and 2010 between the 6 and 48 months of follow-up. However, like the other PROs, there was no statistically significant difference at baseline, or by month 60 (Table 2).

**Fig. 3:**
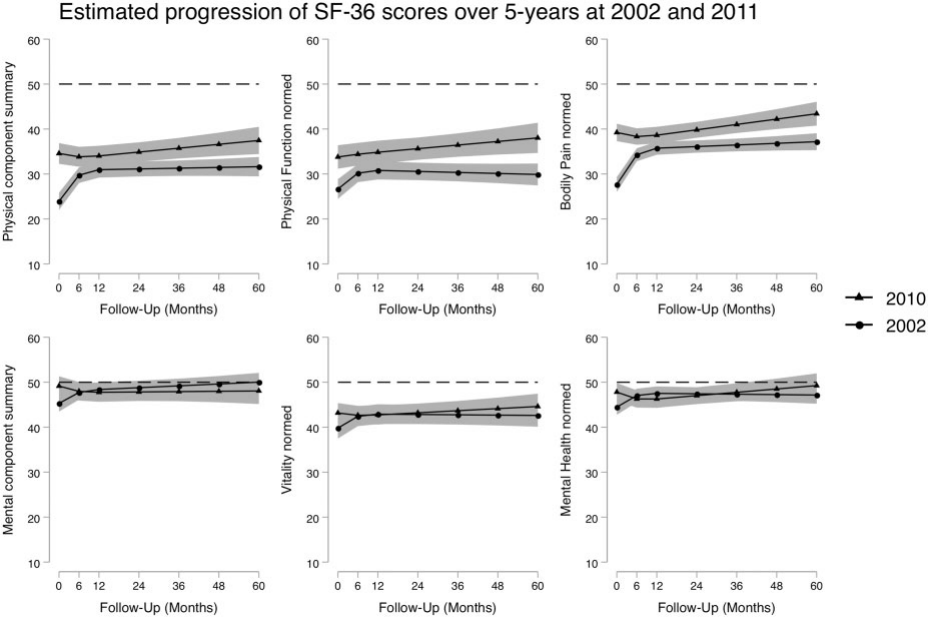
Estimated mean scores of the SF-36 PCS and MCS, along with the Physical Function, Bodily Pain, Vitality and Mental Health sub-domain scores over the first 60 months for patients diagnosed in 2002 and 2010 The black dotted line represents a score of 50, the normalized population average. Patients were diagnosed in 2002 (circle marker) or 2010 (triangle marker). Shaded areas represent the 95% CIs. Models controlled for age at onset, gender, seropositivity status at baseline, baseline BMI, baseline Charlson Comorbidity Index. and use of DMARDs and steroids at baseline assessment. SF-36: Short Form-36; PCS: Physical Component Score; MCS: Mental Component Score.

For those patients in the ERAN cohort where the SF-36 was collected, mixed-effects linear models were used to estimate both the PCS and MCS, as well as the Physical Function, Bodily Pain, Vitality and Mental Health sub-components. As the SF-36 data were only available in ERAN, only 2002 and 2010 recruitment periods were modelled. The progression of these scores are illustrated in Fig. 3, and relative differences between 2010 and 2002 are given in Table 3.

**Table 3:** Estimated differences of the Short-Form 36 at baseline and 6-months at 2010 and 2002

	SF-36 PCS		SF-36 PF		SF-36 BP	
	2010 *vs* 2002		2010 *vs* 2002		2010 *vs* 2002	
	Delta	95% CI	Delta	95% CI	Delta	95% CI
Baseline	10.65***	7.16, 14.14	7.13***	3.17, 11.09	11.58***	8.58, 14.57
Month 60	5.84*	1.36, 10.31	8.14**	3.17, 13.11	6.22**	2.26, 10.17
	SF-36 MCS		SF-36 VT		SF-36 MH	
	2010 *vs* 2002		2010 *vs* 2002		2010 *vs* 2002	
	Delta	95% CI	Delta	95% CI	Delta	95% CI
Baseline	3.86*	0.56, 7.16	3.36*	0.18, 6.54	3.34*	0.25, 6.43
Month 60	−1.96	−6.27, 2.35	1.99	−2.13, 6.11	2.10	−1.93, 6.13

The estimated mean differences, along with their corresponding 95% CI, for the SF-36 PCS and MCS, along with the PF, BP, VT and MH sub-domains at baseline and at month 60, comparing patients diagnosed in 2010 with those diagnosed in 2002. Models controlled for age at onset, gender, seropositivity status at baseline BMI, baseline Charlson Comorbidity Index, and use of DMARDs and steroids at baseline assessment.

* *P* < 0.05,

** *P* < 0.01,

*** *P* < 0.001.

SF-36: Short Form 36; PCS: Physical Component Scale; MCS: Mental Component Scale; PF: Physical Function; BP: Bodily Pain; VT: Vitality; MH: Mental Health.

Relative to 2002, patients diagnosed in 2010 had significantly better PCS at both baseline (*P* < 0.001) and month-60 (*P* < 0.05). This improvement was reflected by similar improvements in both Physical Function and Bodily Pain. However, by month 60, the magnitude of the improvement had reduced. The improvement for patients diagnosed in 2010 was reflected in a greater likelihood of achieving a PCS ≥50 of 21% (OR 0.21; 95% CI 0.13, 0.28) by month 60, compared with those diagnosed in 2002 with just 5% (OR 0.05; 95% CI 0.02, 0.10).

In contrast, whilst patients diagnosed in 2010 experienced significantly better MCS at baseline (*P* < 0.05) relative to those diagnosed in 2002, the magnitude of this difference was much lower. As with the PCS, this difference diminished over time and by month 60 was statistically non-significant. This was reflected in both the Vitality and Mental Health sub-components. The likelihood of achieving a MCS ≥50 units was similar in both 2010 (OR 0.42; 95% CI 0.29, 0.55) and 2002 (OR 0.58; 95% CI 0.49, 0.67).

## Discussion

This study reports significant declines in disease activity in RA over the last two decades, driven largely by reductions in inflammatory markers. However, these improvements in disease activity have not translated into similar levels of improvements for mental health, functional disability, or patient ratings of overall disease activity, pain or vitality/fatigue, where levels have remained relatively stable over the same period. These findings are in keeping with other published work looking at secular changes in PRO [9–12].

Compared with the data presented from the two NOAR cohorts [8], the HAQ trajectories of the 1990 and 2002 cohort follow a similar pattern, albeit starting at a slightly lower score. This is likely due to NOAR including all inflammatory polyarthritic conditions, whereas ERAS and ERAN were restricted to RA patients only. These findings were also corroborated in a recent meta-analysis using data from 29 early RA cohorts (including ERAS and ERAN) of over 10 000 patients, which found that levels of pain, fatigue, physical function and general measures of mental health had not improved when comparing pre- and post-2002 cohorts of patients with early RA. This is despite statistically and clinically meaningful reductions in disease activity levels [31].

Differences in demographic and clinical outcomes at presentation have been examined in this data in more detail by the research team, which found increasing prevalence of comorbid conditions, particularly cardiovascular and non-cardiac vascular morbidities, as well as increases in BMI and age at disease onset [1]. However, despite decreased levels of ESR at presentation, there was little evidence of secular changes in disease activity levels or functional disability at diagnosis. This analysis has developed these findings further to show how these differences at presentation, in conjunction with changes in the treatment of RA, have affected the long-term progression of important RA-related outcomes. Whilst it is likely that adoption of more intensive and aggressive treatment strategies are the primary drivers for the decline in inflammatory markers of the disease [11, 32, 33], it is unclear whether the increases in comorbidities and obesity are hampering equal improvements in pain, fatigue and functional disability.

Using the SF-36 Vitality sub-domain, this study indicated little change in levels of fatigue over time. Data from the British Society for Rheumatology Biologics Register for RA (BSRBR-RA) did indicate statistically and clinically significant reductions in levels of fatigue for those patients treated with anti-TNF-α [34]; however, despite these improvements, there were still a substantial number of patients who achieved disease remission that reported high levels of fatigue [35]. This is suggestive of a heterogeneous RA population, whereby a sub-group of patients are likely to experience increased levels of fatigue over the course of their disease [36].

Previous studies have found little evidence of an association between inflammatory markers and fatigue [37], and any associations with disease activity are likely to be secondary to symptoms of pain and depression [38]. However, there is increasing evidence of a bi-directional relationship between inflammatory markers, such as IL-6 and TNF-α, with depression [39]. The complex relationship between depressive symptoms and inflammation, along with the close associations between depression with pain and fatigue, may in part explain why some patients see improvements in these outcomes with more effective anti-inflammatory therapies [39]. There is a clear need for a conceptual framework that incorporates these complex associations, in order that they be better understood to inform identification and ultimately better interventions [40].

### Strengths and limitations

The use of prospective, observational cohort data allows for the estimation of important outcomes in a true to life, naturally treated, clinical setting. Unlike clinical trials, where patients with high disease levels are typically over-represented, the inclusive nature of the cohorts allows for a wide spectrum of RA patients to be included in the analysis. The emphasis on early RA also allowed for the estimation of the progression of these outcomes at treatment initiation.

However, prospective cohorts are prone to attrition and missing data can hinder the statistical power of longitudinal analysis, particularly when examining group-level differences. The presence of missing data was largely accounted for through the use of mixed models, which utilizes full-information maximum likelihood to estimate missing data using the predictions from observed data in the models.

As ERAS is a historical cohort, it did not collect PGA scores. In order to overcome this, pain scores measured in ERAS using a 0–100 VAS were used. It could be argued that both measures are measuring different constructs: PGA a more overall general health measure, whereas pain VAS specifically targets RA-related pain. Nevertheless, from a statistical point of view, these two measures correlate highly, and demonstrate high levels of agreement. As such, the pain score is likely to be a suitable approximation of the ERAS patients’ PGA score had they been collected routinely.

### Conclusions

The role of improved therapeutic managements has been instrumental in lowering inflammation and reducing the inflammatory aspects of the disease over the last 30 years, the so-called objective markers. However, there is a clear need to examine the subjective aspects of the disease [41], which is driving the discordance between objective measures of inflammation, and the patient-reported measures [42]. Greater incorporation of PRO in both clinical research and practice is vital, as they measure important aspects of the disease not currently assessed in standard disease activity measures [43]. These patient-reported factors have large implications on treatment decisions and call for multi-disciplinary care to address all aspects of patients’ health [41, 44].

Future research should begin to focus on examining possible sub-groups of patients that may progress differently over time, as has already been evidenced with HAQ [6, 45]. Early identification of patient sub-groups who fair worse on pain, fatigue and mental health outcomes, despite improvements in inflammation and general markers of disease activity (so-called ‘persistent symptoms’), would be useful in allocating resources and identifying non-pharmacological therapies targeting these specific aspects of the disease [44].
